# New core-pyrene π structure organophotocatalysts usable as highly efficient photoinitiators

**DOI:** 10.3762/bjoc.9.101

**Published:** 2013-05-07

**Authors:** Sofia Telitel, Frédéric Dumur, Thomas Faury, Bernadette Graff, Mohamad-Ali Tehfe, Didier Gigmes, Jean-Pierre Fouassier, Jacques Lalevée

**Affiliations:** 1 Institut de Science des Matériaux de Mulhouse IS2M – UMR 7361 – UHA; 15, rue Jean Starcky, F-68057 Mulhouse Cedex, France; 2 Aix-Marseille Université, CNRS, Institut de Chimie Radicalaire, UMR 7273, F-13397 Marseille, France; 3 ENSCMu-UHA, 3 rue Alfred Werner, F-68093 Mulhouse Cedex, France

**Keywords:** cationic photopolymerization, free-radical-promoted cationic photopolymerization, photocatalysts, photoinitiators, radical photopolymerization

## Abstract

Eleven di- and trifunctional compounds based on a core-pyrene π structure (Co_Py) were synthesized and investigated for the formation of free radicals. The application of two- and three-component photoinitiating systems (different Co_Pys with the addition of iodonium or sulfonium salts, alkyl halide or amine) was investigated in detail for cationic and radical photopolymerization reactions under near-UV–vis light. The proposed compounds can behave as new photocatalysts. Successful results in terms of rates of polymerization and final conversions were obtained. The strong MO coupling between the six different cores and the pyrene moiety was studied by DFT calculations. The different chemical intermediates are characterized by ESR and laser flash photolysis experiments. The mechanisms involved in the initiation step are discussed, and relationships between the core structure, the Co_Py absorption property, and the polymerization ability are tentatively proposed.

## Introduction

**Scheme 1:**
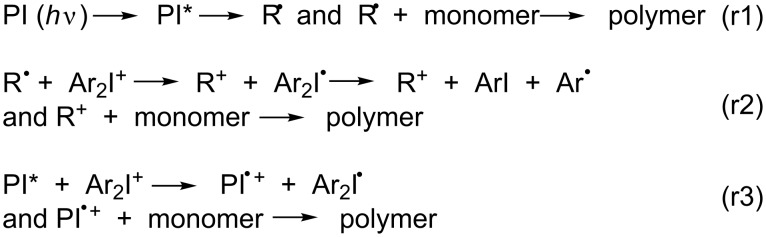
Typical reactions for photoinitiated cationic polymerization.

Free radical sources are encountered in various areas such as organic chemistry, biochemistry and polymer chemistry. In the field of polymer photochemistry applied to photopolymerization reactions, they are referred to as photoinitiators (PI) [1]. These PIs are usable in two scenarios, both of which are light induced. Firstly, they are usable in free radical polymerization (FRP), where the PIs work as either cleavable type I PIs or uncleavable type II PIs in dependence of couples formed by the PI and hydrogen or electron donors (r1 in Scheme 1). Secondly, PIs may be used in free-radical-promoted cationic polymerization (FRPCP), in which the produced radical R^•^ is oxidized by an onium salt, e.g., Ar_2_I^+^, to form Ar_2_I^•^ and a cation R^+^ suitable for the ROP reaction. The Ar_2_I^•^ species is readily decomposed into ArI and Ar^•^ (r2 in Scheme 1). There is also an usual cationic polymerization (CP; r3 in Scheme 1) [1–2]. When the PIs exhibit a catalytic behavior analogous to a photocatalyst (PC) in organic chemistry, they are designed as photoinitiator catalysts (PIC). PIs as well as PICs are based on pure organic, that is, metal free, or organometallic compounds. In the presence of additives, they constitute a photoinitiating system (PIS).

Among others, two points of interest in the photopolymerization area are (i) the reactivity and efficiency of the PIs and PICs and (ii) the irradiation conditions. Indeed, the absorption properties (wavelengths λ, molar extinction coefficients ε), the excited-state processes, the reactivity of the produced radicals, and the characteristics of the light sources (emission spectra, available luminous power density) govern the polymerization efficiency (rate of polymerization, final monomer conversion).

The photopolymerization reactions in radiation curing technologies are often carried out under high-intensity light sources (>1000 mW cm^−2^). Applications in this area and in other fields may require to avoid the use of UV rays delivered by Hg lamps or to irradiate with visible light (400–700 nm) and low photon fluxes (~2–10 mW cm^−2^). Recent works have aimed at working under soft irradiation conditions (near-UV–vis light, low light intensity), which allows the use of Xe lamps, laser diodes, solar radiation, household halogen lamps, fluorescent bulbs and blue, green and white LEDs (see a recent review in [1]).

**Scheme 2:**
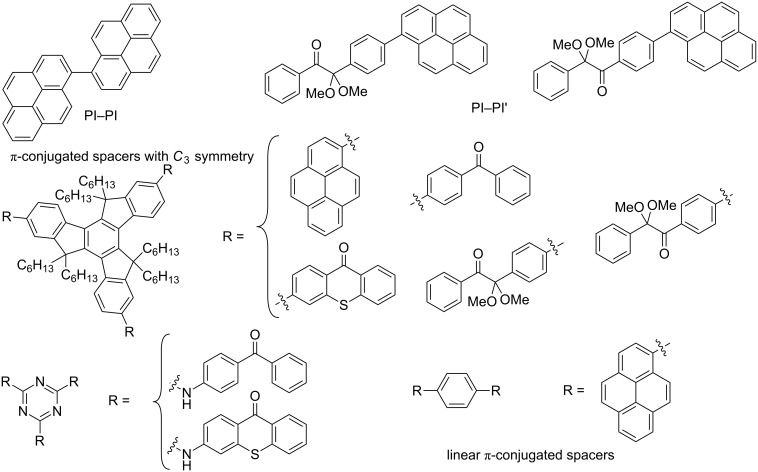
Examples of previously investigated architectures.

New high-performance PIs and PICs are continuously being developed [1–10] through the design of novel structures (e.g., novel chromophores, novel cleavable bonds) and the modification ofexisting structures by using the classical introduction of electron-donating or -withdrawing substituents, conjugated groups or moieties, and more complex changes on the skeleton (see typical recent examples in [11–41]). To meet the challenge of designing efficient PIs and PICs under soft irradiation, we have recently started the search for suitable multifunctional arrangements exhibiting a strong coupling of the molecular orbitals MOs [42–43]. Improved absorption properties (red-shifted λ, higher ε) while keeping a high reactivity have been already achieved. Examples of these investigated architectures (Scheme 2) [42–44] are linked PI–PI units (e.g., PI = pyrene) or PI-PI’ (e.g., PI = pyrene and PI’ = 2,2’-dimethoxy-2-phenylacetophenone) and PI moieties (e.g., PI = benzophenone, thioxanthone, 2,2’-dimethoxy-2-phenylacetophenone, pyrene) linked to a trifunctional core (truxene, triazine, benzene); the same could be expected by using a difunctional core.

**Scheme 3:**
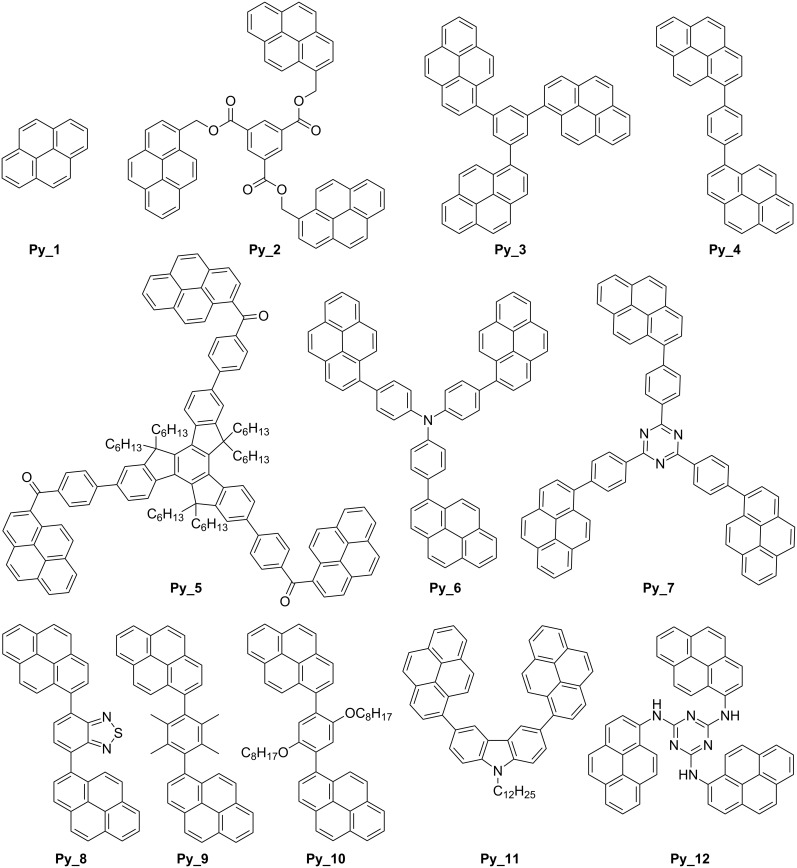
Investigated **Co_Pys**.

**Scheme 4:**
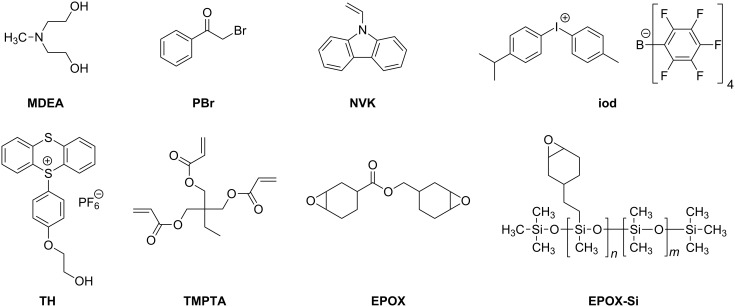
Other chemical compounds.

In the present paper, we consider a series of eleven di- and trifunctional core-pyrene π compounds Co_Py, where the pyrene moiety is used as a PI. The cores include a functionalized truxene (**Py_5**), several substituted phenyl rings (**Py_2**, **Py_3**, **Py_4**, **Py_9**, **Py_10**) two functionalized triazines (**Py_7**, **Py_12**), a triphenylamine (**Py_6**), a carbazole (**Py_11**) and a benzothiadiazole (**Py_8**) (Scheme 3). The idea is to get a high absorption around 380–410 nm where Xe–Hg lamps, Xe lamps, LED, laser diodes and even household halogen lamps are usable. The large choice of Co_Pys should allow for studying the effect of the core on the MO coupling and the resulting absorption properties. The activity of these Co_Py compounds in the FRP of acrylates and the FRPCP (and eventually the cationic polymerization CP) of epoxides through a ring-opening polymerization (ROP) under exposure to the near-UV–vis light delivered by a Hg–Xe lamp (~30 mW cm^−2^) and the visible light of a halogen lamp (soft irradiation conditions; ~10 mW cm^−2^) is investigated. The Co_Pys are used in combination with additives: iodonium or sulfonium salts for ROP and amine or/and alkyl halide for FRP. The monomers investigated are given in Scheme 4. The mechanisms are analyzed by electron spin resonance (ESR), steady-state photolysis, fluorescence and laser flash photolysis (LFP). The ability of Co_Pys to behave as new organophotocatalysts is also discussed.

## Results and Discussion

### Light absorption, redox properties and MO calculations

**Figure 1:**
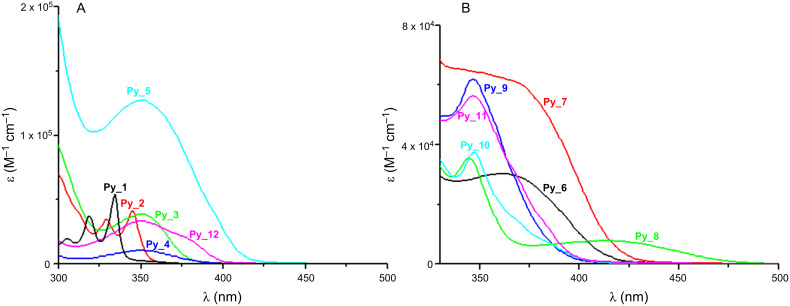
UV–vis absorption spectra of the investigated compounds: (A) In acetonitrile for **Py_1** and acetonitrile/toluene (50/50) for the other molecules, (B) in toluene.

The UV absorption spectra of the different pyrene derivatives are depicted in Figure 1. The solvents were selected for good solubility of the Co_Pys. Absorption maxima are located at ~350 nm for **Py_2**, **Py_3**, **Py_4**, **Py_5**, **Py_12**, **Py_8**, **Py_9**, **Py_10**, **Py_11**, ~370 nm for **Py_6**, **Py_7**, and 330 nm for **Py_1** (Table 1). This corresponds to a red shift of the absorption for **Py_2** to **Py_12** compared to the reference compound **Py_1**. At 365 nm, the maximum emission wavelength of the Hg–Xe lamp, the molar extinction coefficients ε_max_ of the different Co_Pys follow the order **Py_5** > **Py_7** > **Py_11** > **Py_9** > **Py_6** > **Py_12** > **Py_3** > **Py_10** > **Py_8** > **Py_4** > **Py_1** = **Py_2**. The highest ε_max_ is obtained for **Py_5** (ε_353_ = 13 × 10^4^ M^−1^ cm^−1^, ε_346_ = 6 × 10^4^ M^−1^ cm^−1^ versus ε_334_ = 6 × 10^4^ M^−1^ cm^−1^ for **Py_5**, **Py_9** and **Py_1**, respectively). **Py_4** exhibits low ε values, and thus a low efficiency can be expected for this compound.

**Table 1:** Properties of the pyrene derivatives in acetonitrile (*) and toluene. Light absorption and emission properties (experimental λ_max,abs,exp_ and calculated λ_max,abs,calc_ maximum absorption wavelengths, emission wavelengths λ_max,em_), excited-state energy levels (*E*_S_), oxidation potentials (*E*_ox_) and free-energy changes Δ*G* (see text) [1].

	λ_max,abs,exp_ (nm)	λ_max,abs,calc_^a^ (nm)	λ_max,em_ (nm)	*E*_ox_ (V/SCE)	*E*_S_ (eV)	Δ*G*_PBr_ (eV)	Δ*G*_Iod_ (eV)

Py_1^*^	334	327 (f = 0.27)	393	1.33	3.44	−1.33	−1.9
**Py_2**	344	334 (f = 0.41)	380	0.8	3.45	−1.87	−2.45
**Py_3**	351	356 (f = 0.50)	393	0.9	3.36	−1.68	−2.26
**Py_4**	351	373 (f = 0.3)	423	0.6	3.21	−1.83	−2.41
**Py_5**	353	387 (f = 1.16)	404	0.7	3.24	−1.76	−2.34
**Py_6**	365	344 (f = 0.52); 403 (f = 0.74)	441	0.9	3.04	−1.36	−1.94
**Py_7**	367	344 (f = 0.68)	440	–	3.02	–	–
**Py_8**	344	365 (f = 0.39)	530	0.7	2.6	−1.12	−1.7
**Py_9**	346	336 (f = 0.44)	428	0.8	3.2	−1.62	−2.2
**Py_10**	347	376 (f = 0.51)	425	0.9	3.2	−1.52	−2.1
**Py_11**	346	370 (f = 0.4)	409	−	3.22	–	–
**Py_12**	346	377 (f = 1.00)	397	0.6	3.22	−1.84	−2.42

^a^Molecular orbital MO calculations (using the time-dependent density functional theory at B3LYP/6-31G* level on the relaxed geometries calculated at UB3LYP/6-31G* level); f = oscillator strength.

The oxidation potentials of the Co_Pys (Table 1) range from 1.33 V (**Py_1**) to 0.6 V (**Py_4**, **Py_12**). The singlet-state energy is also affected by the core of the structure, i.e., 2.6 eV for **Py_8** versus 3.44 eV for the reference **Py_1**. The free-energy change Δ*G* of the Co_Py/phenacyl bromide PBr (or the iodonium salt, Iod) interaction is very favorable (Δ*G* << 0). **Py_2** exhibits the most favorable Δ*G*s: −2.45 eV (Iod) and −1.87 eV (PBr).

**Figure 2:**
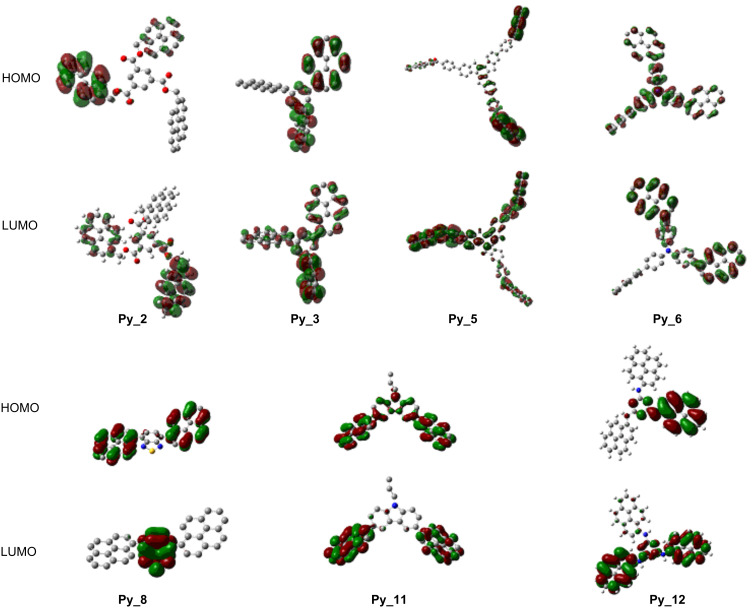
HOMO–LUMO orbitals for **Py_2**, **Py_3**, **Py_5**, **Py_6**, **Py_8**, **Py_11** and **Py_12** involved in the π–π* transition (calculated at UB3LYP/6-31G* level; for **Py_5** and **Py_11**, the alkyl chains have been simplified to reduce the computational cost).

MO calculations show that the calculated and experimental values (Table 1) are approximately the same, e.g., a difference of 5 to 10 nm is observed for **Py_2** and **Py_3**. The oscillator strengths (noted f in Table 1) are higher for the new proposed structures than for **Py_1** in agreement with their better light absorption properties (Figure 1). A strong coupling of the π MOs of the core with those of the pyrene substituents is shown supporting a clear ππ* character of the HOMO–LUMO transition, as evidenced in Figure 2 for the investigated derivatives. For the various π-core/pyrene substituent combinations, different coupling strengths can be observed (Figure 2). Strong couplings of the MOs lead to red-shifted transitions compared to **Py_1** and enhanced absorption coefficients (Figure 1), e.g., for **Py_3**, **Py_5**, **Py_6**, **Py_8**, **Py_11** and **Py_12**. A strong delocalization of the HOMOs and LUMOs is observed in line with their better absorption properties. For **Py_2**, the coupling is weak and the absorption properties are moderately affected compared to **Py_1**.

### Photochemical reactivity

#### Fluorescence experiments

**Figure 3:**
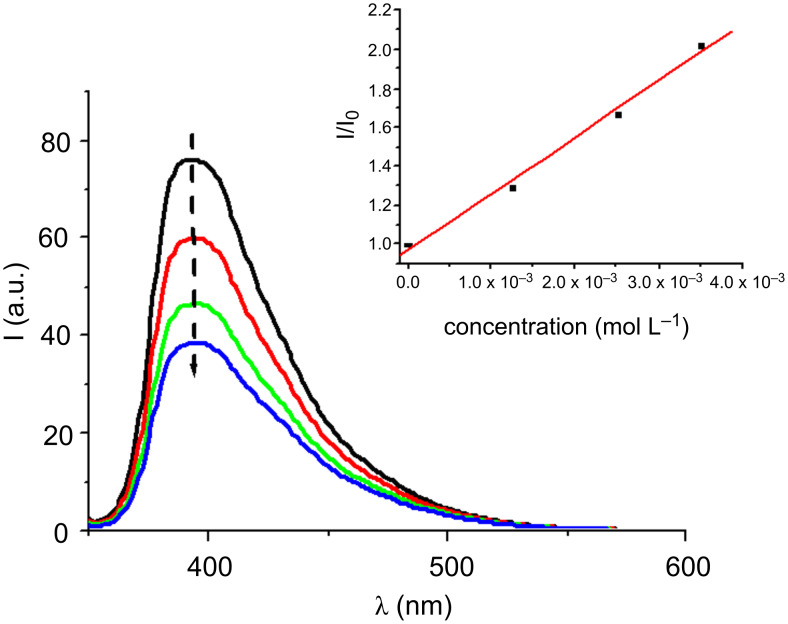
Fluorescence quenching of ^1^**Py_3** by the phenacylbromide (PBr) in acetonitrile/toluene (50/50). Insert: Stern–Volmer plot (the concentrations used are given in this plot).

**Scheme 5:**
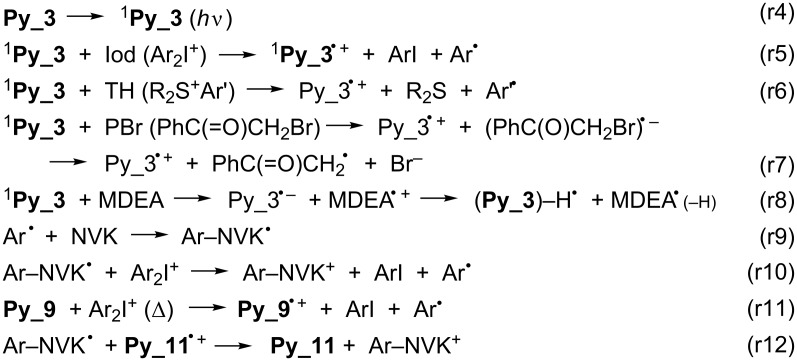
Photochemical processes for the different Co_Pys.

A strong quenching of the Co_Py singlet states by PBr, amine (MDEA), Iod and the sulfonium salt TH is found. **Py_3** is selected for this mechanistic study (Figure 3) because of the high reactivity of this compound in photopolymerization experiments (see below). The rate constants in that case are 1.5 × 10^10^, 9 × 10^9^, 1 × 10^10^ and 3.7 × 10^9^ M^−1^ s^−1^, respectively. The ^1^**Py_3**/PBr, ^1^**Py_3**/Iod and ^1^**Py_3**/TH interactions are diffusion-controlled in full agreement with the highly favorable Δ*G*s (Table 1; the reduction potential for TH is −1.3 V [1]; the calculated Δ*G* is −1.16 eV). They correspond to an efficient electron transfer followed by the usual fast generation of radicals from the radical anion species (r5–r7 in Scheme 5) or an electron/proton transfer with the amine (r8 in Scheme 5).

#### ESR spin trapping experiments

**Figure 4:**
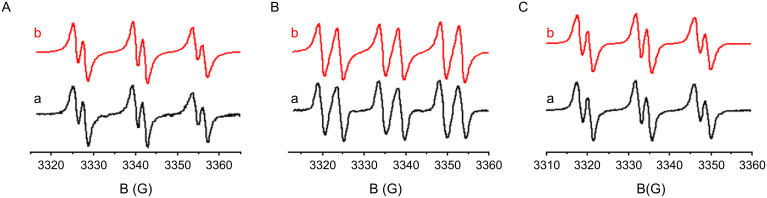
ESR spectra obtained upon irradiation of (A) **Py_3**/Iod, (B) **Py_3**/PBr and (C) **Py_3**/EDB in *tert*-butylbenzene. Phenyl-*N*-*tert-*butylnitrone (PBN) is used as a spin trap; (a) experimental and (b) simulated ESR spectra.

In ESR spin trapping experiments upon UV light exposure, the interaction of the Co_Pys with the iodonium salt Iod (Figure 4A) or the sulfonium salt TH (Figure 1 in Supporting Information File 1) leads to an aryl radical Ar^•^ (e.g., hyperfine coupling constants *hfcs* of the PBN adduct: a_N_ = 14.2 G and a_H_ = 2.2 G in agreement with known data [22–25,45]) in line with (r5). In the same way, phenacyl radicals and α-aminoalkyl radicals (r7 and r8 in Scheme 5) are observed in ^1^**Py_3**/PBr and ^1^**Py_3**/EDB (a_N_ = 14.6 G, a_H_ = 4.5 G and a_N_ = 14.3 G, a_H_ = 2.5 G, respectively; Figure 4B and Figure 4C; ethyldimethylaminobenzoate (EDB) instead of MDEA is used to avoid a high polarity of the sample preventing an ESR investigation). The **Py_3**/Iod or the **Py_3**/TH couple generates a cation radical (**Py_3^•^**^+^) that can initiate a cationic polymerization whereas the phenacyl and aminoalkyl radicals formed in **Py_3**/PBr or **Py_3**/MDEA are susceptible to radical polymerization.

#### Laser flash photolysis

Upon laser excitation of **Py_3** at 355 nm, a triplet state T_1_ characterized by a maximum absorption at ~420 nm and a rather long lifetime is formed (*t* > 4 μs) (Figure S2 in Supporting Information File 1). This T_1_ state is similar to that of **Py_1** [31]. The short S_1_ lifetime (e.g., ~55 ns under N_2_ for **Py_3**) and the diffusion controlled interactions of S_1_ with Iod, PBr or MDEA (see above) prevent a significant production of T_1_ in Co_Py/Iod (PBr or MDEA). Therefore, a triplet-state pathway can be neglected in the investigated systems.

#### Thermal processes

**Figure 5:**
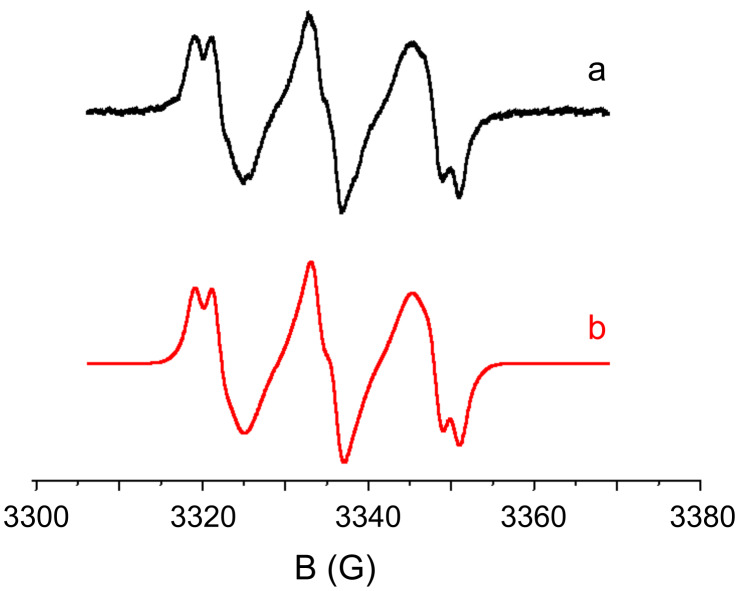
ESR-spin trapping spectra of **Py_9**/Iod in *tert*-butylbenzene (storage at rt for 24 h); (a) experimental and (b) simulated spectra.

For some Co_Py/Iod couples, a slow thermal redox process can also occur. Indeed, in ESR–ST experiments, aryl radicals are generated without irradiation within 24 h (no free radical was observed in **Py_9** or Iod alone). For example, in **Py_9**/Iod, the same radicals as in r5 are produced (Figure 5; *hfcc*s: a_N_ = 14.2 G and a_H_ = 2.2 G for the PBN radical adduct). Peroxyl radicals are also observed, as aryls easily add to O_2_ (a_N_ = 13.3 G and a_H_ = 1.6 G for the PBN radical adduct, in agreement with [45]). As in r5 in Scheme 5, a cation radical **Py_9^•^**^+^ is concomitantly formed. This thermal process is slow as *E*_ox_(**Py_9**) > *E*_red_(Iod) (Table 1). The formation of radical cations can be worthwhile to initiate cationic polymerization at room temperature (see below).

### Ability of the different pyrene structures in photopolymerization reactions

#### Cationic photopolymerization (CP)

**Figure 6:**
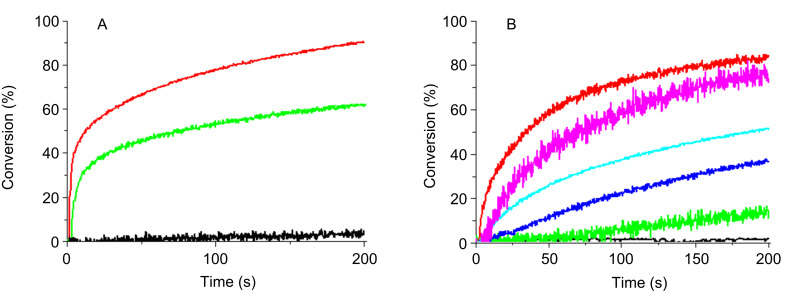
Photopolymerization profiles of EPOX upon Xe–Hg lamp irradiation (λ > 340 nm) under air for different photoinitiating systems: (A) (a) Iod (2% w/w); (b) **Py_3**/Iod (1%/2% w/w); (c) **Py_1**/Iod (1%/2% w/w); (B) (a) TH (2% w/w); (b) **Py_3**/TH (0.2%/2% w/w); (c) **Py_8**/TH (0.2%/2% w/w); (d) **Py_9**/TH (0.2%/2% w/w); (e) **Py_10**/TH (0.2%/2% w/w); (f) **Py_11**/TH (0.2%/2% w/w).

**Figure 7:**
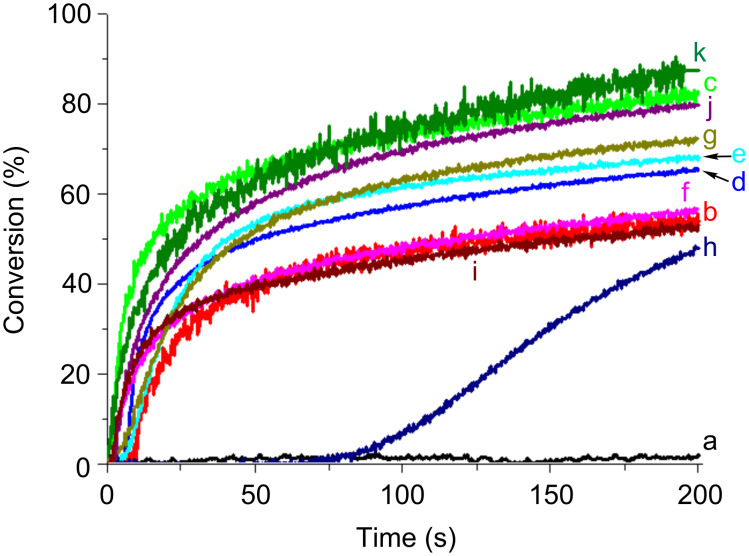
Photopolymerization profiles of EPOX upon a Xe–Hg lamp irradiation (λ > 340 nm) under air for different photoinitiating systems: (a) Iod (2% w/w); (b) **Py_1**/Iod (0.2%/2% w/w); (c) **Py_3**/Iod (0.2%/2% w/w); (d) **Py_4**/Iod (0.2%/2% w/w); (e) **Py_5**/Iod (0.2%/2% w/w); (f) **Py_6**/Iod (0.2%/2% w/w); (g) **Py_7**/Iod (0.2%/2% w/w); (h) **Py_8**/Iod (0.2%/2% w/w); (i) **Py_9**/Iod (0.2%/2% w/w); (j) **Py_10**/Iod (0.2%/2% w/w) ; (k) **Py_11**/Iod (0.2%/2% w/w).

**Figure 8:**
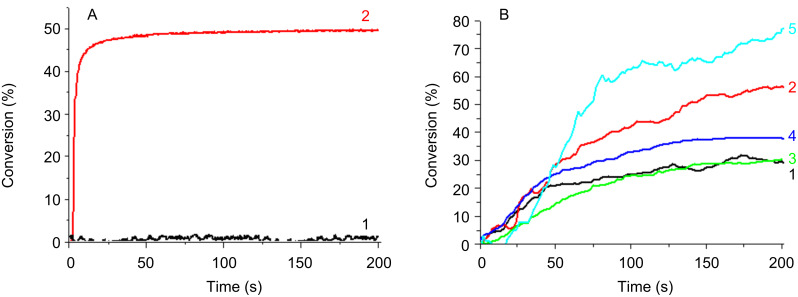
(A) Photopolymerization profiles of EPOX-Si upon a Xe–Hg lamp irradiation (λ > 340 nm) under air for different photoinitiating systems: (A) (1) Iod (2% w/w); (2) **Py_3/**Iod (1%/2% w/w); (B) Photopolymerization profiles of EPOX under air upon halogen-lamp irradiation in the presence of (1) **Py_6**/Iod (0.2%/2% w/w); (2) **Py_7**/Iod (0.2%/2% w/w); (3) **Py_10**/Iod (0.2%/2% w/w); (4) **Py_11**/Iod (0.2%/2% w/w); (5) **Py_11**/Iod/NVK (0.2%/2%/2% w/w).

The ring-opening polymerization profiles of EPOX upon exposure to the Xe–Hg lamp under air using two-component PISs are shown in Figure 6, Figure 7 and Figure 8. A comparison of the photopolymerization profiles using the **Py_1** (to **Py_12**)/Iod couples (Figures 6–8) shows that the fastest polymerization rates (Rp) and the highest final conversion (conv) are obtained with **Py_11**/Iod and **Py_3**/Iod. No polymerization is observed in the presence of Iod alone Figure 6A and Figure 8A, curves a.) **Py_3**/Iod leads to a conv ~90% (Figure 6A, curve b) compared to **Py_1**/Iod (conv ~50%; Figure 6A, curve c). Almost all the new proposed structures are better than the starting structure **Py_1** (Figure 7, curve b). This highlights the interest of the present approach associated with the modification of the Py chromophore. CP can also be initiated in the presence of the sulfonium salt TH, e.g., with **Py_3**, **Py_8**, **Py_9**, **Py_10** and **Py_11** (Figure 6B). The conversions reach 80% and 75% with **Py_3**/TH and **Py_11**/TH, respectively (after 200 s light exposure). EPOX-Si can also be easily polymerized (Figure 8). **Py_3**/Iod (Figure 8A) is still efficient (conv ~50%).

In these systems, the Co_Py^•+^ generated in r5,r6 (Scheme 5) can initiate the CP process. The relative efficiency of these Co_Py/Iod (or TH) combinations will be affected by different parameters: (i) their relative light absorption properties, (ii) the quantum yields of radical or radical cation formation in the singlet state (the singlet state is predominant, see above), (iii) the rate constants for reactions r5 and r6 and (iv) the ability of Co_Py^•+^ to initiate a ring-opening polymerization process. Structure–reactivity relationships for the different derivatives can hardly be extracted. This is probably ascribed to a strong interplay between (i) to (iv).

A cationic photopolymerization profile of EPOX under very soft irradiation (halogen lamp exposure) is represented in Figure 8B. In the presence of Iod, several Co_Pys exhibiting an acceptable absorption at λ > 380 nm lead to efficient polymerization reactions. For example, conv = 40 and 55% within 200 s with the **Py_7**/Iod and **Py_11**/Iod couples.

The three-component PIS (**Py_11**/Iod/NVK) (Figure 8B, curve 5) allows an increase of the EPOX conversion up to 75%. A consumption of the NVK double bond is observed (Figure S3 in Supporting Information File 1). As known in other systems, this is accounted for by the addition of Ar^•^ to the NVK double bond (formation of an Ar–NVK^•^ radical) (r9 in Scheme 5). This electron rich radical is easily oxidized (r10 in Scheme 5) by Iod, the resulting cation Ar–NVK^+^ being able to initiate the cationic polymerization.

#### Thermal polymerization at room temperature

Remarkably, the thermal polymerization of EPOX can also be initiated by different Co_Py/Iod (1%/2% w/w) systems. This reaction proceeds smoothly at rt but is completed after 24 h, e.g., with **Py_9**, **Py_6**, **Py_10** or **Py_11**/Iod, the final conversion is >55%. As supported by the ESR experiments (see above), the presence of, e.g., **Py_9^•^**^+^ explains the thermal initiation of the cationic polymerization of the epoxide (r11 in Scheme 5). For the other Co_Py, a good thermal stability is found. This dual behavior of some Co_Pys, which are able to initiate both a thermal and a photochemical cationic polymerization, can be very useful to initiate the polymerization in the shadow areas.

#### Free radical photopolymerization (FRP)

**Figure 9:**
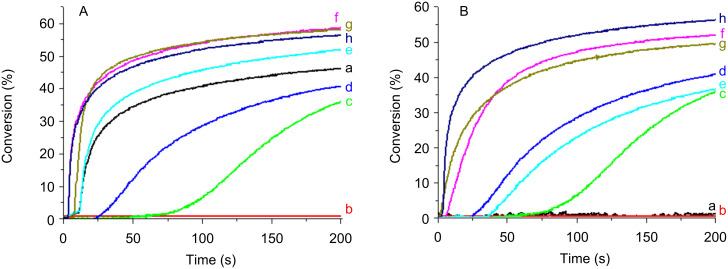
Photopolymerization profiles of TMPTA upon Xe–Hg lamp irradiation (λ > 340 nm) in laminate for different photoinitiating systems: (A) (a) PBr (2% w/w); (b) **Py_2** (0.2% w/w); (c) **Py_3** (0.2% w/w); (d) **Py_5** (0.2% w/w); (e) **Py_2**/PBr (0.2%/2% w/w); (f) **Py_3**/PBr (0.2%/2% w/w); (g) **Py_5**/PBr (0.2%/2% w/w); (h) **Py_3**/MDEA/PBr (0.2%/2%/2% w/w); (B) (a) MDEA (2% w/w); (b) **Py_2** (0.2% w/w); (c) **Py_3** (0.2% w/w); (d) **Py_5** (0.2% w/w); (e) **Py_2**/MDEA (0.2%/2% w/w); (f) **Py_3**/MDEA (0.2%/2% w/w); (g) **Py_5**/MDEA (0.2%/2% w/w); (h) **Py_3**/MDEA/PBr (0.2%/2%/2% w/w).

Typical free radical polymerization profiles of TMPTA upon the Xe–Hg lamp exposure in laminate by using the Co_Py/MDEA (or PBr) two-component PISs are represented in Figure 9. In the presence of Co_Py alone, FRP occurs but the inhibition time is high and the final conversion is low (50 s; 35% with **Py_3**). The addition of PBr (Figure 9A) or MDEA (Figure 9B) shortens the inhibition time and increases the final conversion to ~55%. For the Co_Py/amine or Co_Py/PBr two-component systems, the radicals generated in r7 and r8 in Scheme 5 can initiate the FRP process. Due to its absorption in the UV, PBr alone initiates the acrylate photopolymerization (conv = 45%; Figure 9A, curve a). No striking improvement of the final conversion is observed when using the **Py_3**/MDEA/PBr three-component PIS instead of **Py_3**/PBr (~55%; Figure 9A, curve f versus h), but both Rp and conv are better when comparing to **Py_3**/MDEA.

#### Photocatalyst behavior of the Co_Pys

**Figure 10:**
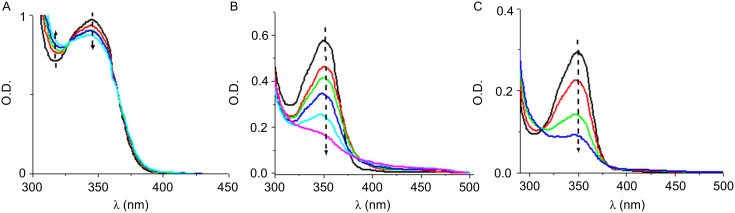
Photolysis of (A) the **Py_3**/PBr couple, (B) the **Py_3**/Iod couple, and (C) the **Py_3**/MDEA couple; Xe–Hg lamp irradiation. In acetonitrile/toluene (50/50); from *t* = 0 to 5 min.

The photocatalytic behavior of the Co_Pys is investigated in the most interesting compounds reported above for the photopolymerization reactions. The steady-state photolysis of **Py_3**/PBr, **Py_3**/Iod, **Py_3**/MDEA couples highlights the consumption of the pyrene moiety under light exposure (Figure 10). In **Py_3**/PBr and **Py_3**/MDEA, isosbestic points are present at 330 nm (Figure 10A) and 315 nm (Figure 10B), respectively, suggesting stoichiometric reactions and no other side reactions.

**Figure 11:**
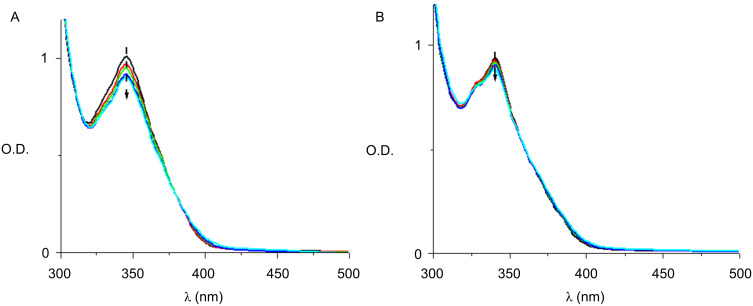
Photolysis of (A) the **Py_11**/Iod and (B) the **Py_11**/Iod/NVK couple. Halogen lamp irradiation. In acetonitrile/toluene (50/50); from *t* = 0 to 5 min.

**Scheme 6:**
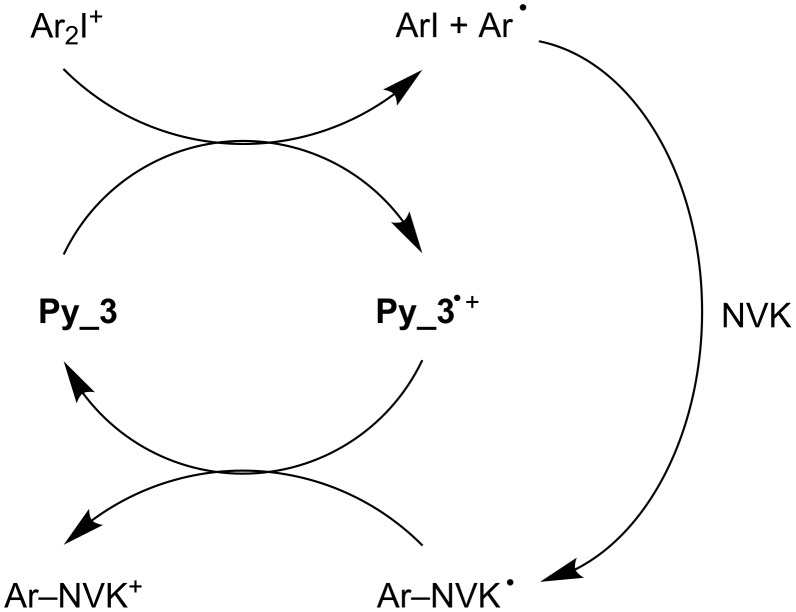
The oxidative cycle.

The addition of NVK to **Py_11**/Iod shows that the photolysis is faster with **Py_11**/Iod than with **Py_11**/Iod/NVK couples (Figure 11A versus Figure 11B). This difference highlights that in the presence of NVK, **Py_11** is regenerated according to an oxidation cycle (Scheme 6). Therefore, **Py_11** behaves as a new photocatalyst in agreement with (r12 in Scheme 5).

**Scheme 7:**
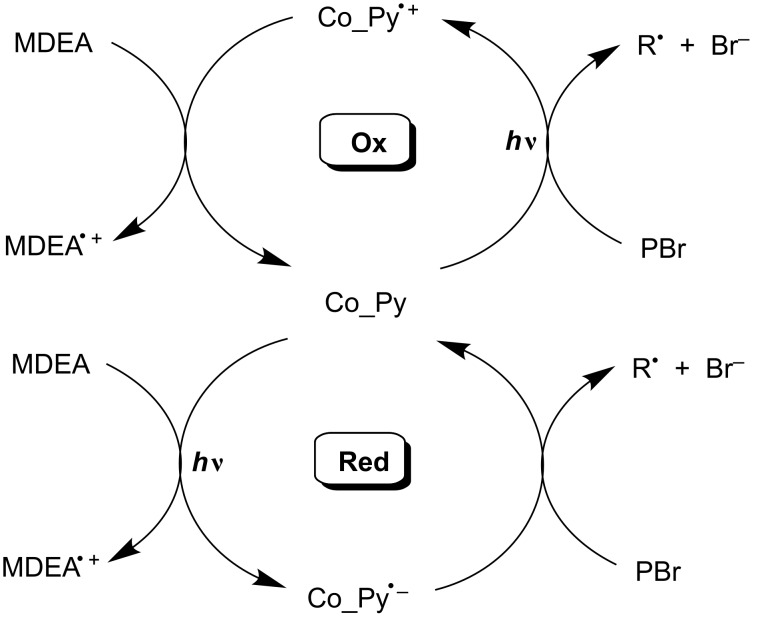
Oxidation versus reduction cycles.

In the Co_Py/PBr/MDEA three-component system, reactions (r7, r8) are probably competitive (e.g., k[MDEA] ~ k[PBr] using **Py_3**). As a consequence, both a reduction and an oxidation cycle could simultaneously proceed (Scheme 7). However, the ESR and polymerization results (see above) (i) suggest that the contribution of the reduction cycle should be weak and (ii) confirm that the regeneration in the oxidation cycle has a low efficiency. This might be due to a MDEA^•+^ → MDEA_–H_^•^ process (in r8 in Scheme 5) less efficient than PBr^•−^ → phenacyl radical (in r7 in Scheme 5). Nevertheless, such an oxidation cycle that can contribute here to some extent appears as one of the rare examples observed in photocatalyst (PC)/PBr/amine systems, because most of them work according to a reduction cycle [30,46] (see other examples in [47]; in a related system, still unpublished, based on another PC, we have demonstrated the true occurrence of a unique oxidation cycle).

## Conclusion

In this paper, the core-pyrene π structures Co_Pys appear as interesting photoinitiators. Successful cationic and radical photopolymerization reactions were carried out under near-UV–vis irradiation. A photocatalytic behavior is noted, that is, an oxidation cycle for Co_Py/Iod/NVK and a partial oxidation cycle for Co_Py/MDEA/PBr. These experimentally and theoretically investigated di- and trifunctional compounds outline the interest in a suitable strong MO coupling. This could open the way to an a priori choice of absorption spectra driven by the theoretical design of chemical compounds. Other proposals will be provided in the future.

## Experimental

### Co_Pys

The synthesized compounds are presented in Scheme 3. The procedures are presented in detail in Supporting Information File 1. ^1^H and ^13^C NMR spectra and HRMS mass spectral analysis are also given. **Py_1** was purchased from Aldrich. 1,4-Di(pyren-1-yl)benzene (**Py_4**) was prepared by adapting a literature procedure [48]. **Py_12** was synthesized as previously reported [49]. The syntheses of **Py_2** [50], **Py_3** [51], **Py_6** [52] and **Py_7** [53] were already reported under reaction conditions different to the ones reported in this article and in lower yields. The results obtained in our work agree with the previously reported characterization of these products.

### Other chemical compounds

The [methyl-4-phenyl (methyl-1-ethyl)-4-phenyl] iodonium tetrakis(pentafluorophenyl)borate [54–55] (Iod; PI 2074 from Bluestar Silicones - France) was used as the iodonium salt (Scheme 4). Phenacyl bromide (PBr), *N*-methyldiethanolamine (MDEA) and *N*-vinylcarbazole (NVK) were obtained from Aldrich and (4-hydroxyethoxyphenyl) thianthrenium hexafluorophosphate (TH; recrystallized form of Esacure 1187) from Lamberti Spa. The (epoxycyclohexylethyl)methylsiloxane-dimethylsiloxane copolymer (EPOX-Si) was obtained from Bluestar Silicones-France (Silcolease UV POLY 200); trimethylolpropane triacrylate TMPTA and (3,4-epoxycyclohexane)methyl 3,4-epoxycyclohexylcarboxylate (EPOX or UVACURE 1500) were provided by Cytec.

### Photopolymerization procedures

TMPTA was irradiated at room temperature under lamination conditions: the formulation (25 μm thick) is sandwiched between two polypropylene films. The weight percentages for the photoinitiators are given in the figure captions. The evolution of the acrylate content was continuously followed by real-time FTIR spectroscopy (FTIR NEXUS 870) at ~1620 cm^−1^. The formulations (25 μm thick) based on the cationic monomers (EPOX, EPOX-Si) deposited on a BaF_2_ pellet were irradiated under air inside the IR spectrometer cavity at room temperature [56–57]. The evolution of the epoxy content was also continuously followed by real-time FTIR spectroscopy (FTIR NEXUS 870). A Xe–Hg lamp (Hamamatsu, L8252, 150 W, filtered light at λ > 340 nm; intensity ~ 30 mW/cm²) and a halogen lamp (intensity ~10 mW/cm²; the emission spectrum is given in [43]) were used as the irradiation sources.

### ESR experiments

ESR spin-trapping (ESR-ST) experiments were carried out by using a X-Band ESR spectrometer (EMX-plus from Bruker Biospin or MS400 from Magnettech). The radicals were produced at RT under Xe–Hg lamp exposure and trapped by phenyl-*N*-*tert-*butylnitrone (PBN) according to a procedure described in detail in [58–59].

### Redox potentials

The redox potentials were measured in acetonitrile by cyclic voltammetry with tetrabutylammonium hexafluorophosphate (98%) as a supporting electrolyte (0.1 M) (Voltalab 6 Radiometer; the working electrode was a platinum disk and the reference a saturated calomel electrode-SCE). Ferrocene was used as a standard and the potentials were determined from half-peak potentials. The free energy change Δ*G*_et_ for an electron-transfer reaction is calculated from the classical Rehm–Weller equation (Equation 1) [60] where *E*_ox_, *E*_red_, *E*_S_ and *C* are the oxidation potential of the donor, the reduction potential of the acceptor, the excited state energy and the coulombic term for the initially formed ion pair, respectively. *C* is neglected as usually done in polar solvents.

[1]
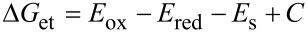


### Fluorescence experiments

The fluorescence properties of the different Co_Pys were studied by using a JASCO FP-750 spectrometer.

### Computational procedure

Molecular orbital calculations were done with the Gaussian 03 suite of programs [61–62]. The electronic absorption spectra for the different compounds were calculated with the time-dependent density functional theory at B3LYP/6-31G* level on the relaxed geometries calculated at UB3LYP/6-31G* level.

### Laser flash photolysis

Nanosecond laser flash photolysis (LFP) experiments were carried out [26–35] by using a Qswitched nanosecond Nd/YAG laser (λ_exc_ = 355 nm, 9 ns pulses; energy reduced down to 10 mJ) from Continuum (Minilite) and an analyzing system consisting of a ceramic Xenon lamp, a monochromator, a fast photomultiplier, and a transient digitizer (Luzchem LFP 212).

## Supporting Information

File 1 Experimental procedures, characterization data, and additional spectra.

## References

[1] Fouassier J-P, Lalevée J (2012). Photoinitiators for Polymer Synthesis: Scope, Reactivity and Efficiency.

[2] Fouassier J-P (1995). Photoinitiation, Photopolymerization, Photocuring.

[3] Fouassier J-P, Rabek F J (1993). Radiation Curing in Polymer Science and Technology.

[4] Crivello J V, Dietliker K, Bradley G (1999). Photoinitiators for Free Radical, Cationic and Anionic Photopolymerization.

[5] Dietliker K A (2002). Compilation of Photoinitiators Commercially Available for UV Today.

[6] Belfied K D, Crivello J V (2003). Photoinitiated Polymerization.

[7] Allen N S (2010). Photochemistry and Photophysics of Polymer Materials.

[8] Scranton A B, Bowman A, Peiffer R W (1997). Photopolymerization: Fundamentals and Applications.

[9] Green W A (2010). Industrial Photoinitiators.

[10] Mishra M K, Yagci Y (2008). Handbook of Vinyl Polymers.

[11] Wang K, Jiang S, Liu J, Nie J, Yu Q (2011). Prog Org Coat.

[12] Temel G, Enginol B, Aydin M, Karaca Balta D, Arsu N (2011). J Photochem Photobiol, A: Chem.

[13] Wei J, Wang B (2011). Macromol Chem Phys.

[14] Asvos X, Siskos M G, Zarkadis A K, Hermann R, Brede O (2011). J Photochem Photobiol, A: Chem.

[15] Kabatc J, Jurek K (2012). Polymer.

[16] Rosspeintner A, Griesser M, Pucher N, Iskra K, Liska R, Gescheidt G (2009). Macromolecules.

[17] Karaka-Balta D, Temel G, Goksu G, Okal N, Arsu N (2012). Macromolecules.

[18] Yilmaz G, Acik G, Yagci Y (2012). Macromolecules.

[19] Kumbaraci V, Aydogan B, Talinli N, Yagci Y (2012). J Polym Sci, Part A: Polym Chem.

[20] Tunc D, Yagci Y (2011). Polym Chem.

[21] Kreutzer J, Dogan Demir K, Yagci Y (2011). Polym J.

[22] Lalevée J, Blanchard N, Tehfe M A, Morlet-Savary F, Fouassier J P (2010). Macromolecules.

[23] Zhang G, Song I Y, Ahn K H, Park T, Choi W (2011). Macromolecules.

[24] Lalevée J, Peter M, Dumur F, Gigmes D, Blanchard N, Tehfe M-A, Morlet-Savary F, Fouassier J P (2011). Chem–Eur J.

[25] Tehfe M-A, Ma L, Graff B, Morlet-Savary F, Fouassier J-P, Zhao J, Lalevée J (2012). Macromol Chem Phys.

[26] Lalevée J, Blanchard N, Fries C, Tehfe M A, Morlet-Savary F, Fouassier J P (2011). Polym Chem.

[27] Tehfe M-A, Zein-Fakih A, Lalevée J, Dumur F, Gigmes D, Graff B, Morlet-Savary F, Hamieh T, Fouassier J P (2013). Eur Polym J.

[28] Telitel S, Lalevée J, Blanchard N, Kavalli T, Tehfe M-A, Schweizer S, Morlet-Savary F, Graff B, Fouassier J P (2012). Macromolecules.

[29] Tehfe M-A, Lalevée J, Morlet-Savary F, Graff B, Blanchard N, Fouassier J P (2012). ACS Macro Lett.

[30] Tehfe M-A, Lalevée J, Morlet-Savary F, Graff B, Blanchard N, Fouassier J P (2012). Macromolecules.

[31] Tehfe M-A, Dumur F, Contal E, Graff B, Morlet-Savary F, Gigmes D, Fouassier J P, Lalevée J (2013). Polym Chem.

[32] Tehfe M-A, Lalevée J, Morlet-Savary F, Graff B, Fouassier J-P (2012). Macromolecules.

[33] Lalevée J, Dumur F, Tehfe M-A, Zein-Fakih A, Gigmes D, Morlet-Savary F, Fouassier J P (2012). Polymer.

[34] Lalevée J, Tehfe M-A, Dumur F, Gigmes D, Blanchard N, Morlet-Savary F, Graff B, Fouassier J P (2012). ACS Macro Lett.

[35] Tehfe M-A, Lalevée J, Telitel S, Sun J, Zhao J, Graff B, Morlet-Savary F, Fouassier J-P (2012). Polymer.

[36] Aydogan B, Yagci Y, Toppare L, Jockusch S, Turro N J (2012). Macromolecules.

[37] Aydogan B, Durmaz Y Y, Kahveci M U, Uygun M, Tasdelen M A, Yagci Y (2011). Macromol Symp.

[38] Aydogan B, Gunbas G E, Durmus A, Toppare L, Yagci Y (2010). Macromolecules.

[39] Bulut U, Gunbas G E, Toppare L (2010). J Polym Sci, Part A: Polym Chem.

[40] Bulut U, Balan A, Caliskan C (2011). J Polym Sci, Part A: Polym Chem.

[41] Yagci Y, Jockusch S, Turro N J (2007). Macromolecules.

[42] Lalevée J, Tehfe M A, Dumur F, Gigmes D, Graff B, Morlet-Savary F, Fouassier J P (2013). Macromol Rapid Commun.

[43] Tehfe M-A, Dumur F, Graff B, Clément J-L, Gigmes D, Morlet-Savary F, Fouassier J P, Lalevée J (2013). Macromolecules.

[44] Tehfe M-A, Lalevée J, Telitel S, Contal E, Dumur F, Gigmes D, Bertin D, Nechab M, Graff B, Morlet-Savary F (2012). Macromolecules.

[45] Fisher H (2005). Magnetic Properties of Free Radicals, Nitroxide Radicals and Nitroxide Based High-Spin Systems. Landolt-Börnstein - Group II, Molecules and Radicals.

[46] Narayanam J M R, Stephenson C R J (2011). Chem Soc Rev.

[47] Nguyen J D, Tucker J W, Konieczynska M D, Stephenson C R J (2011). J Am Chem Soc.

[48] Yang C-H, Guo T-F, Sun I-W (2007). J Lumin.

[49] Tehfe M-A, Dumur F, Graff B, Morlet-Savary F, Fouassier J-P, Gigmes D, Lalevée J (2012). Macromolecules.

[50] Rajakumar P, Visalakshi K, Ganesan S, Maruthamuthu P, Suthanthiraraj S A (2012). Bull Chem Soc Jpn.

[51] Lee D-H, Jin M-J (2011). Org Lett.

[52] Kim M K, Kwon J, Hong J P, Lee S, Hong J I (2011). Bull Korean Chem Soc.

[53] Ishi-i T, Yaguma K, Thiemann T, Yashima M, Ueno K, Mataka S (2004). Chem Lett.

[54] Castellanos F, Fouassier J P, Priou D, Cavezzan A (1997). Onium borates/borates of organometallic complexes and cationic initiation of polymerization therewith. U.S. Patent.

[55] Castellanos F, Fouassier J P, Priou C, Cavezzan J (1996). J Appl Polym Sci.

[56] Tehfe M-A, Lalevée J, Gigmes D, Fouassier J P (2010). Macromolecules.

[57] Tehfe M-A, Lalevée J, Gigmes D, Fouassier J P (2010). J Polym Sci, Part A: Polym Chem.

[58] Tordo P, Gilbert B C, Atherton N M, Davies M J (1998). Spin-trapping: recent developments and applications. Electron Pragmagnetic Resonance.

[59] Lalevée J, Dumur F, Mayer C R, Gigmes D, Nasr G, Tehfe M-A, Telitel S, Morlet-Savary F, Graff B, Fouassier J P (2012). Macromolecules.

[60] Rehm D, Weller A (1970). Isr J Chem.

[61] (2003). Gaussian 03.

[62] Foresman J B, Frisch A (1996). Exploring Chemistry with Electronic Structure Methods.

