# Supplementary material for: New core-pyrene π structure organophotocatalysts usable as highly efficient photoinitiators
Source: Beilstein J Org Chem. 2013 May 7;9:877–90. doi: 10.3762/bjoc.9.101 (PMC3678508; doi:10.3762/bjoc.9.101)
Supplement: File 1 — Experimental procedures, characterization data, and additional spectra. [file Beilstein_J_Org_Chem-09-877-s001.pdf]

# Supporting Information

# for

# New core-pyrene π structure organophotocatalysts usable as highly efficient photoinitiators

Sofia Telitel[1], Frédéric Dumur[2], Thomas Faury[2], Bernadette Graff[1], Mohamad-Ali Tehfe[1], Didier Gigmes*[2], Jean-Pierre Fouassier[3] and Jacques Lalevée*[1]

Address: [1]Institut de Science des Matériaux de Mulhouse IS2M – UMR 7361 – UHA; 15, rue Jean Starcky, F-68057 Mulhouse Cedex, France, [2]Aix-Marseille Université, CNRS, Institut de Chimie Radicalaire, UMR 7273, F-13397 Marseille, France and [3]Former address ENSCMu-UHA, 3 rue Alfred Werner, F-68093 Mulhouse Cedex, France.

Email: Jaques Lalevée - jacques.lalevee@uha.fr and Didier Gigmes - didier.gigmes@univ-amu.fr

*Corresponding author

## Experimental procedures, characterization data, and additional spectra

**Synthetic procedures for the Co_Pys:**

All reagents and solvents were purchased from Aldrich or Alfa Aesar and used as received. All reactions were performed under argon unless otherwise stated. Mass spectrometry was performed by the Spectropole of Aix-Marseille University. ESI mass spectra were recorded with a 3200 QTRAP (Applied Biosystems SCIEX) mass spectrometer. The HRMS mass spectral analysis was performed with a QStar Elite (Applied Biosystems SCIEX) mass spectrometer. Elemental analyses were recorded with a Thermo Finnigan EA 1112 elemental analysis apparatus driven by the Eager 300 software. $^{1}H$ and $^{13}C$ NMR spectra were determined at room temperature in 5 mm o.d. tubes on a Bruker Avance 400 spectrometer of the Spectropole: $^{1}H$ (400 MHz) and $^{13}C$ (100 MHz). The $^{1}H$ chemical shifts were referenced to the solvent peak $CDCl_3$ (7.26 ppm), DMSO-$d_6$ (2.49 ppm) and the $^{13}C$ chemical shifts were referenced to the solvent peak $CDCl_3$ (77 ppm), DMSO-$d_6$ (49 ppm). 1,4-Dibromo-2,3,5,6-tetramethylbenzene [1], 1,4-di-(*n*-octoxy)benzene [2] and (2,3,5,6-tetramethyl-1,4-phenylene)diboronic acid [3] were synthesized as previously reported. (4-Bromophenyl)(pyren-1-yl)methanone (**2**) was synthesized by two procedures different from those previously reported [4]. 2,7,12-Tris(4,4,5,5-tetramethyl-1,3,2-dioxaborolan-2-yl)hexahexyltruxene was prepared following a procedure previously reported in the literature without modification and with similar yields [5].

Synthesis of *tris*(pyren-1-ylmethyl) benzene-1,3,5-tricarboxylate (**Py_2**).

1 g (4.76 mmol) of 1,3,5-benzenetricarboxylic acid were suspended in 10 mL of thionyl chloride, and a few drops of DMF were added. The solution was heated under reflux overnight. After cooling, the solvent was removed under reduced pressure, furnishing the trimesic chloride (1.26 g, quantitative yield). The carboxylic acid chloride was suspended in DCM (10 mL) and triethylamine (1.45 g, 1.9 mL, 3 equiv) was added. This solution was slowly added to a solution of pyrene 1-methanol (3.31 g, 3 equiv) and the mixture was stirred under reflux overnight. After cooling, the formed precipitate was filtered off and washed several times with chloroform. The solvent was removed under reduced pressure and the residue was purified by column chromatography ($SiO_2$) by using a gradient of eluent from pentane/DCM 1:1 to methanol. The title product was isolated as a light yellow solid in 78% yield (3.17 g). $^{1}H$ NMR ($CDCl_3$) δ (ppm): 6.10 (s, 6H, $CH_2$), 7.99-8.22 (m, 23H), 8.34 (d, 4H, $J$ = 9.2 Hz), 8.82 (d, 3H, $J$ = 1.6 Hz); $^{13}C$ NMR ($CDCl_3$) δ (ppm): 65.8, 122.7, 124.6, 125.6, 126.1, 127.3, 127.8, 128.3, 129.7, 130.6, 131.1, 131.3, 131.9, 134.5, 134.7, 164.9; HRMS–ESI (*m*/*z*): theor: 853.2585 found: 853.2588 ($[M + H]^+$ detected).

Synthesis of 1,3,5-tri(pyren-1-yl)benzene (**Py_3**).

To a solution of pyreneboronic acid (2.5 g, 10.16 mmol) and 1,3,5-tribromobenzene (1 g, 3.18 mmol) in THF (80 mL), was added $Pd(PPh_3)_4$ (60 mg) and aqueous KOH (5 mL, 2 M). The mixture was heated under reflux for 48 h under a nitrogen atmosphere. Then the mixture was poured into water, and the aqueous layer was extracted with $CHCl_3$. The combined organic phases were washed with brine and dried ($MgSO_4$). The residue was purified by column chromatography ($SiO_2$) by using DCM/acetone 1:1 as the eluent. During evaporation, a light brown solid formed, which was filtered off, washed with pentane, and dried under vacuum. 1.90 g of the photoinitiator was obtained in 88% yield. $^1H$ NMR ($CDCl_3$) δ (ppm): 8.02 (t, 3H, $J$ = 7.6 Hz), 8.09-8.15 (m, 12H), 8.20 (t, 6H, $J$ = 7.0 Hz), 8.25-8.30 (m, 6H), 8.61 (d, 3H, $J$ = 9.3 Hz); $^{13}C$ NMR ($CDCl_3$) δ (ppm): 124.8, 124.9, 125.1, 125.20, 120.22, 126.0, 127.4, 127.6, 127.8, 127.9, 128.6, 130.8, 131.0, 131.5, 131.7, 137.2, 141.5; HRMS–ESI (*m*/*z*): theor: 678.2348 found: 678.2344 ($[M]^+$ detected).

Synthesis of 1,4-di(pyren-1-yl)benzene (**Py_4**).

First procedure: To a solution of 1,4-benzenediboronic acid (1 g, 6.03 mmol,) and 1-bromopyrene (5.1 g, 18.1 mmol, 3 equiv) in THF (80 mL), was added $Pd(PPh_3)_4$ (60 mg) and aqueous KOH (5 mL, 2 M). The mixture was heated under reflux for five days under a nitrogen atmosphere. Then the mixture was poured into water, and the aqueous layer was extracted with $CHCl_3$. The combined organic phases were washed with brine and dried ($MgSO_4$). During evaporation, a light yellow solid formed, which was filtered off, washed several times with pentane, and dried under vacuum. 2.65 g of the title compound were obtained in 92% yield.

Second procedure: To a solution of 1-pyreneboronic acid (2 g, 8.33 mmol), 1,4-dibromobenzene (0.64 g, 2.78 mmol,) in 80 mL dry THF was added 0.2 g of $Pd(PPh_3)_4$, followed by aqueous KOH (5 mL, 2 M). The solution was heated under reflux for two days. Then the mixture was poured into water, and the aqueous layer was extracted with $CHCl_3$. The combined organic phases were washed with brine and dried ($MgSO_4$). During evaporation, a light yellow solid formed, which was filtered off, washed several times with pentane, and dried under vacuum. 1.18 g of the title compound was obtained in 89% yield. $^1H$ NMR ($CDCl_3$) δ (ppm): 7.85 (s, 4H), 8.02-8.24 (m, 14H), 8.30 (d, 2H, $J$ = 7.8 Hz), 8.40 (d, 2H, $J$ = 9.3 Hz); $^{13}C$ NMR ($CDCl_3$) δ (ppm): 124.8, 124.9, 125.0, 125.1, 125.2, 125.4, 126.1, 127.48, 128.53, 127.6, 127.7, 128.6, 130.6, 130.7, 131.1, 131.6; HRMS–ESI (*m*/*z*): theor: 478.1722 found: 478.1719 ($[M]^+$ detected).

Synthesis of (4-bromophenyl)(pyren-1-yl)methanol (**1**).

*First procedure*: 1.83 g (6.50 mmol) of 1-bromopyrene was dissolved in 100 mL dry THF. The solution was cooled to −78 °C and 2.86 mL (7.15 mmol, 2.5 M in hexanes, 1.1 equiv) of *n*-BuLi was slowly added. The reaction was stirred for 30 min and 1.20 g (6.50 mmol) of 4-bromobenzaldehyde was added. The reaction was allowed to warm to room temperature and stirring was maintained overnight. The solution was quenched with aqueous HCl (6 N) and stirring was maintained for 10 min. Water was added and the solution was extracted with chloroform 3 times, the organic phases were combined, dried over magnesium sulfate and the solvent removed under reduced pressure. The residue was purified by column chromatography ($SiO_2$) using DCM/acetone 1:1 as the eluent. During evaporation, a precipitate formed, which was filtered off, washed with pentane, and dried under vacuum. 1.64 g of the title product was obtained in 65% yield.

*Second procedure*: 2 g (8.48 mmol) of 1,4-dibromobenzene was dissolved in 50 mL dry THF and the solution was cooled to 0 °C. 4.1 mL of *n*-BuLi (2.5 M in hexanes, 1.2 equiv) was slowly added and stirring was maintained for 15 min and pyrene 1-carbaldehyde (1.95 g, 8.48 mmol) in 15 mL dry THF was slowly added. The reaction mixture was allowed to warm to room temperature and stirring was maintained for 48 h. The solution was quenched with aqueous HCl (6 N) and stirring was maintained for 10 min. Water was added and the solution was extracted with chloroform 3 times, the organic phases were combined, dried over magnesium sulfate, and the solvent removed under reduced pressure. The residue was purified by column chromatography ($SiO_2$) using DCM/acetone 1:1 as the eluent. 1.38 g of the title product was obtained in 42% yield. $^1$H NMR ($CDCl_3$) δ (ppm): 2.83 (brs, 1H, OH), 6.69 (s, 1H), 7.25 (d, 2H, $J$ = 8.5 Hz), 7.39 (d, 2H, $J$ = 8.5 Hz), 7.95-8.04 (m, 5H), 8.10-8.20 (m, 4H); $^{13}$C NMR ($CDCl_3$) δ (ppm): 72.8, 121.4, 122.7, 124.6, 124.8, 124.9, 125.2, 125.4, 126.0, 127.3, 127.5, 127.87, 127.93, 128.5, 130.4, 131.0, 131.2, 131.48, 131.54, 135.8, 142.4; HRMS–ESI (*m*/*z*): 387.0379 theor: found: 387.0376 ($[M + H]^+$ detected).

Synthesis of (4-bromophenyl)(pyren-1-yl)methanone (**2**).

To a solution of 616 mg (1.59 mmol) of alcohol **1** in 10 mL of $CH_2Cl_2$ was added 515 mg (2.39 mmol) of PCC. The mixture was stirred at room temperature for 5 h. The solution was directly subjected to column chromatography ($SiO_2$) using DCM as the eluent. The ketone was eluted as the first band. The title compound was isolated as a light yellow solid (410 mg, 67% yield). $^1$H NMR ($CDCl_3$) δ (ppm): 7.62 (d, 2H, $J$ = 8.4 Hz), 7.76 (d, 2H, $J$ = 8.4 Hz),

8.05 (td, 3H, *J* = 9.4 Hz, *J* = 1.9 Hz), 8.11 (t, 1H, *J* = 8.4 Hz), 8.17-8.19 (m, 2H), 8.24 (t, 2H, *J* = 8.3 Hz), 8.33 (d, 1H, *J* = 9.3 Hz); $^{13}$C NMR ($CDCl_3$) δ (ppm): 123.7, 124.3, 124.5, 124.7, 126.0, 126.2, 126.4, 126.8, 127.1, 128.4, 129.0, 129.3, 129.7, 130.6, 131.1, 131.8, 132.0, 132.3, 133.2, 137.5, 197.2; HRMS (ESI MS) *m*/*z*: theor: 385.0223 found: 385.0226 ($[M + H]^+$ detected).

Synthesis of **Py_5**.

To a solution of (4-bromophenyl)(pyren-1-yl)methanone (**2**) (1.23 g, 3.2 mmol) and 2,7,12-*tris*(4,4,5,5-tetramethyl-1,3,2-dioxaborolan-2-yl)hexahexyltruxene (980 mg, 0.8 mmol) in THF (80 mL), was added $Pd(PPh_3)_4$ (60 mg) and aqueous KOH solution (5 mL, 2 M). The mixture was heated under reflux for 48 h under nitrogen. Then the mixture was poured into water and the aqueous layer was extracted with $CHCl_3$. The combined organic phases were washed with brine and dried ($MgSO_4$). The residue was purified by column chromatography ($SiO_2$) using DCM/acetone 1:1 as the eluent. During evaporation a light brown solid formed, which was filtered off, washed with pentane, and dried under vacuum. 1.35 g of photoinitiator was obtained in 96% yield. $^{1}$H NMR ($CDCl_3$) δ (ppm): 0.52-0.59 (m, 18H), 0.84-0.91 (m, 48H), 2.16-2.22 (m, 6H), 2.98-3.05 (m, 6H), 7.74-7.77 (m, 6H), 7.87 (d, 6H, *J* = 8.4 Hz), 8.04-8.09 (m, 9H), 8.12-8.28 (m, 21H), 8.42 (d, 3H, *J* = 9.3 Hz), 8.47 (d, 3H, *J* = 8.4 Hz); $^{13}$C NMR ($CDCl_3$) δ (ppm): 13.9, 22.3, 24.0, 29.5, 31.5, 37.1, 55.9, 120.8, 123.9, 124.5, 124.8, 124.9, 125.1, 125.5, 125.9, 126.1, 126.4, 126.8, 127.1, 127.3, 128.9, 129.1, 129.7, 130.7, 131.8, 132.0, 133.0, 133.4, 137.3, 137.8, 138.0, 140.5, 145.8, 146.0, 154.5, 198.0; HRMS–ESI (*m*/*z*): theor: 1758.9707 found:1758.9712 ($[M]^+$ detected).

Synthesis of tris(4-(pyren-1-yl)phenyl)amine (**Py_6**).

To a solution of 1-pyreneboronic acid (2 g, 8.13 mmol), tris(4-bromophenyl)amine (0.98 g, 2.03 mmol) in 80 mL dry THF was added $Pd(PPh_3)_4$ (0.2 g) followed by aqueous KOH (5 mL, 2 M). The solution was heated under reflux for two days. Then the mixture was poured into water and the aqueous layer was extracted with $CHCl_3$. The combined organic phases were washed with brine and dried ($MgSO_4$). During evaporation, a light yellow solid formed, which was filtered off, washed several times with pentane, and dried under vacuum (1.46 g, 85% yield). $^{1}$H NMR ($CDCl_3$) δ (ppm): 7.54 (d, 6H, *J* = 8.5 Hz), 7.65 (d, 6H, *J* = 8.5 Hz), 7.98-8.08 (m, 14H), 8.15-8.24 (m, 10H), 8.36 (d, 3H, *J* = 9.3 Hz); $^{13}$C NMR ($CDCl_3$) δ (ppm): 124.2, 124.7, 124.8, 125.0, 125.1, 125.2, 125.4, 126.0, 127.3, 127.4, 127.5, 127.6,

127.7, 128.5, 130.5, 131.1, 131.6, 131.7, 146.9; HRMS–ESI (*m*/*z*): 846.3155 theor: found: 846.3152 ($[M + H]^+$ detected).

Synthesis of 2,4,6-*tris*(4-(pyren-1-yl)phenyl)-1,3,5-triazine (**Py_7**).

To a solution of 1-pyreneboronic acid (2 g, 8.13 mmol), 2,4,6-tris(4-bromophenyl)-1,3,5-triazine (1.11 g, 2.03 mmol) in 80 mL dry THF was added $Pd(PPh_3)_4$ (0.2 g) followed by aqueous KOH (5 mL, 2 M). The solution was heated under reflux for two days. Then the mixture was poured into water. An insoluble brown-yellow precipitated formed, which was filtered off, washed several times with THF, acetone and DCM. 1.68 g of the title product was obtained in 91% yield. $^1H$ NMR (DMSO-$d_6$) δ (ppm): 7.50-7.63 (m, 12H), 8.02-8.54 (m, 27H); HRMS–ESI (*m*/*z*): 909.3144 theor: found: 909.3140 ($[M]^+$ detected); Anal. calcd for $C_{69}H_{39}N_3$: C, 91.1; H, 4.3; N, 4.6; found: C, 91.4; H, 4.6; N, 4.4%.

Synthesis of 4,7-di(pyren-1-yl)benzo[*c*][1,2,5]thiadiazole (**Py_8**).

To a solution of 1-pyreneboronic acid (2 g, 8.33 mmol), 4,7-dibromobenzo[*c*][1,2,5]thiadiazole (0.82 g, 2.78 mmol) in 80 mL dry THF was added $Pd(PPh_3)_4$ (0.2 g) followed by aqueous KOH (5 mL, 2 M). The solution was heated under reflux for two days. During that time, a light red precipitate formed. After cooling, the precipitate was filtered off, washed with DCM, THF and acetone. 1.30 g of a red solid was isolated in 87% yield. $^1H$ NMR (DMSO-$d_6$) δ (ppm): 8.06-8.08 (m, 8H), 8.19-8.38 (m, 12H); HRMS–ESI (*m*/*z*): 537.1420 theor: found: 537.1422 ($[M]^+$ detected); Anal. calcd for $C_{38}H_{20}N_2S$: C, 85.0; H, 3.8; N, 5.2; found: C, 85.1; H, 3.6; N, 5.4%.

Synthesis of 1,1'-(2,3,5,6-tetramethyl-1,4-phenylene)dipyrene (**Py_9**).

This product was synthesized according to two procedures.

*First procedure*: To a solution of 1-pyreneboronic acid (2 g, 8.33 mmol), 1,4-dibromo-2,3,5,6-tetramethylbenzene (0.81 g, 2.78 mmol) in 80 mL dry THF was added $Pd(PPh_3)_4$ (0.2 g) followed by aqueous KOH (5 mL, 2 M). The solution was heated under reflux for two days during which time a precipitate formed, which was filtered off, washed with THF, and dried under vacuum. 252 mg of the product were obtained in 17% yield.

*Second procedure*: To a solution of (2,3,5,6-tetramethyl-1,4-phenylene)diboronic acid (0.5 g, 2.25 mmol), bromopyrene (1.90 g, 6.76 mmol) in 80 mL dry THF was added $Pd(PPh_3)_4$ (0.1 g) followed by aqueous KOH (5 mL, 2 M). The solution was heated under reflux for three days during which time a precipitate formed, which was filtered off, washed with THF,

and dried under vacuum. 373 mg of the title compound were obtained in 31% yield. $^1$H NMR (DMSO-$d_6$) δ (ppm): 2.48 (s, 12H), 7.52-7.65 (m, 4H), 8.03 (d, 2H, *J* = 9.3 Hz), 8.13 (t, 2H, *J* = 7.5 Hz), 8.20 (d, 2H, *J* = 7.8 Hz), 8.29 (d, 2H, *J* = 8.0 Hz), 8.34 (d, 2H, *J* = 4.9 Hz), 8.40 (d, 2H, *J* = 8.4 Hz), 8.53 (d, 2H, *J* = 7.8 Hz); HRMS–ESI (*m*/*z*): 534.2348 theor: found: 534.2350 ([M]$^{+\cdot}$detected); Anal. calcd for $C_{42}H_{30}$: C, 94.3; H, 5.7; found: C, 94.7; H, 5.5%.

Synthesis of 1,1'-(2,5-*bis*(octyloxy)-1,4-phenylene)dipyrene (**Py_10**).

To a solution of 1-pyreneboronic acid (2 g, 8.33 mmol), 1,4-dibromo-2,5-bis(octyloxy)benzene (1.37 g, 2.78 mmol) in 80 mL dry THF was added 0.2 g of $Pd(PPh_3)_4$ followed by aqueous KOH (5 mL, 2 M). The solution was heated under reflux for two days. Then the mixture was poured into water. The aqueous layer was extracted with $CHCl_3$ and the combined organic phase was washed with brine and dried ($MgSO_4$). During evaporation, a light yellow solid formed, which was filtered off, washed several times with pentane, and dried under vacuum (1.80 g, 88% yield). $^1$H NMR ($CDCl_3$) δ (ppm): 0.72 (t, 6H, *J* = 7.6 Hz), 0.82-0.95 (m, 16H), 1.00-1.03 (m, 4H), 1.32-1.42 (m, 4H), 3.83 (m, 4H, *J* = 6.5 Hz), 7.22 (s, 2H), 7.98-8.28 (m, 18H); $^{13}$C NMR ($CDCl_3$) δ (ppm):14.0, 22.5, 25.8, 29.0, 29.1, 29.2, 31.6, 69.8, 118.08, 118.11, 124.5, 124.88, 124.91, 124.96, 125.0, 125.9, 126.3, 127.1, 127.2, 127.3, 127.5, 128.3, 129.37, 129.40, 130.7, 130.82, 130.84, 131.5, 134.5, 150.9; HRMS–ESI (*m*/*z*): 735.4197 theor: found: 735.4198 ([M]$^{+\cdot}$ detected).

Synthesis of 3,6-dibromo-9-dodecyl-9*H*-carbazole (**3**)

3,6-dibromo-9*H*-carbazole (4 g, 12.30 mmol) was suspended in 30 mL DMF, potassium hydroxide (3.45 g, 61.54 mmol, 5 equiv) was added, and stirring was maintained at room temperature for 1 h. Then, 1-bromododecane (3.68 g, 3.54 mL, 14.76 mmol, 1.2 equiv) was added and the solution was vigorously stirred at 50 °C overnight. The reaction mixture was poured onto ice and the aqueous phase was extracted with AcOEt several times. The organic phases were combined, dried over magnesium sulfate, and the solvent removed under reduced pressure. The residue was purified by column chromatography ($SiO_2$) by using a gradient of eluent from DCM/pentane 1:1 to DCM. 5.58 g of a white solid were obtained (92% yield). $^1$H NMR ($CDCl_3$) δ (ppm): 0.76 (t, 3H, *J* = 6.5 Hz), 0.95-1.25 (m, 18H), 1.56 (qt, 2H, *J* = 6.2 Hz), 3.90 (t, 2H, *J* = 6.8 Hz), 6.97 (d, 2H, *J* = 8.7 Hz), 7.32 (dd, 2H, *J* = 8.7 Hz, *J* = 1.6 Hz), 7.84 (d, 2H, *J* = 1.6 Hz); $^{13}$C NMR ($CDCl_3$) δ (ppm): 14.0, 22.5, 25.5, 27.0, 29.17, 29.20, 29.3, 29.4, 29.5, 31.8, 43.0, 110.1, 111.7, 122.9, 123.1, 128.7, 138.9; HRMS–ESI (*m*/*z*): theor: 492.0896 found: 492.0895 ([M + H]$^+$ detected).

Synthesis of 9-dodecyl-3,6-di(pyren-1-yl)-9*H*-carbazole (**Py_11**).

To a solution of 1-pyreneboronic acid (2 g, 8.33 mmol), 3,6-dibromo-9-dodecyl-9*H*-carbazole (1.34 g, 2.78 mmol) in 80 mL dry THF was added 0.2 g of $Pd(PPh_3)_4$ followed by aqueous KOH (5 mL, 2 M). The solution was heated under reflux for two days. Then the mixture was poured into water and the aqueous layer was extracted with $CHCl_3$. The combined organic phases were washed with brine and dried ($MgSO_4$). During evaporation, a light yellow solid formed, which was filtered off, washed several times with pentane, and dried under vacuum. $^1$H NMR ($CDCl_3$) δ (ppm): 0.76 (t, 3H, $J$ = 6.9 Hz), 0.95-1.45 (m, 18H), 1.88-1.92 (m, 2H), 4.28 (t, 2H, $J$ = 7.3 Hz), 7.32-8.25 (m, 24H); $^{13}$C NMR ($CDCl_3$) δ (ppm): 14.2, 22.7, 27.5, 29.3, 29.4, 29.5, 29.6, 29.7, 30.4, 32.0, 108.7, 110.7, 122.3, 122.5, 123.1, 124.7, 124.9, 125.1, 125.3, 125.7, 127.2, 127.3, 127.4, 128.3, 128.4, 128.6, 130.3, 130.6, 131.1, 131.6, 132.2, 137.8, 138.7, 139.4, 140.3, 141.3; HRMS (ESI MS) $m/z$: theor: 736.3938 found: 736.3940 ($[M + H]^+$ detected).

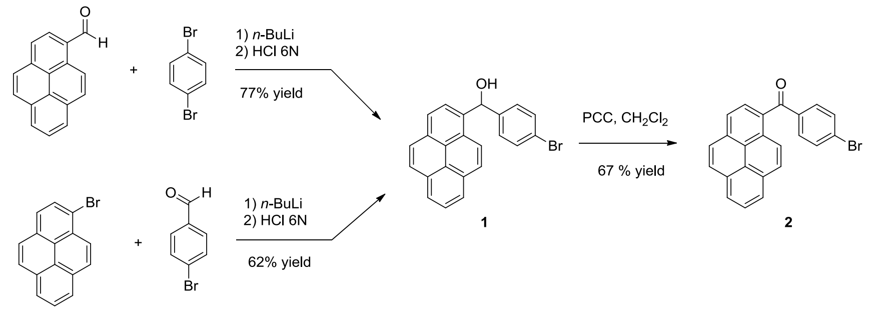


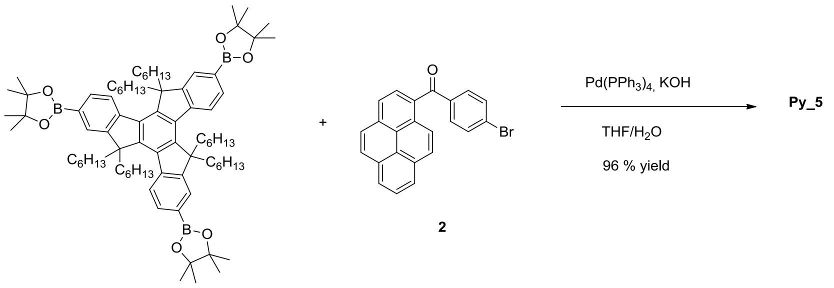


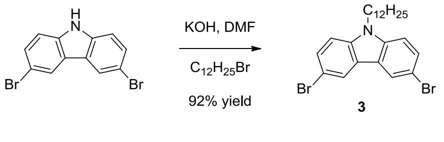

## References


[1] I. Kaur, M. Jazdzyk, N. N. Stein, P. Prusevich, G. P. Miller, *J. Am. Chem. Soc.*, 2010, 132, 1261-1263.

[2] J.Y. Lin, Z.-Z. Jin, Y. Xia, Z.-Y. Zhou, X. Wu, D.-X. Zhu, Z.-M. Su, *Polymer*, 2007, 48, 4028-4033

[3] T. Faury, F. Dumur, S. Clair, M. Abel, L. Porte, D. Gigmes, *CrystEngComm*, 2013, submitted.

[4] M. Beinhoff, W. Weigel, M. Jurczok, W. Rettig, C. Modrakowski, I. Brüdgam, H. Hartl, A. D. Schlüter, *Eur. J. Org. Chem.* 2001, 3819-3829.

[5] J.L. Wang, Y.T. Chan, C.N. Moorefield, J. Pei, D.A. Modarelli, N.C. Romano, G.R. Newkome, *Macromol. Rapid Commun*. 2010, 31, 850-855.


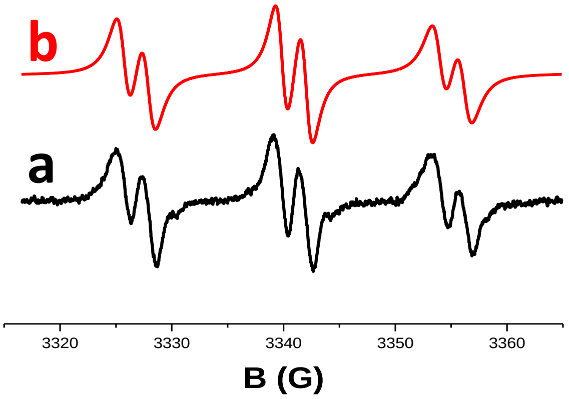


**Figure S1:** Photolysis of the Py_**3**/TH couple; UV lamp irradiation. In acetonitrile/toluene (50/50).

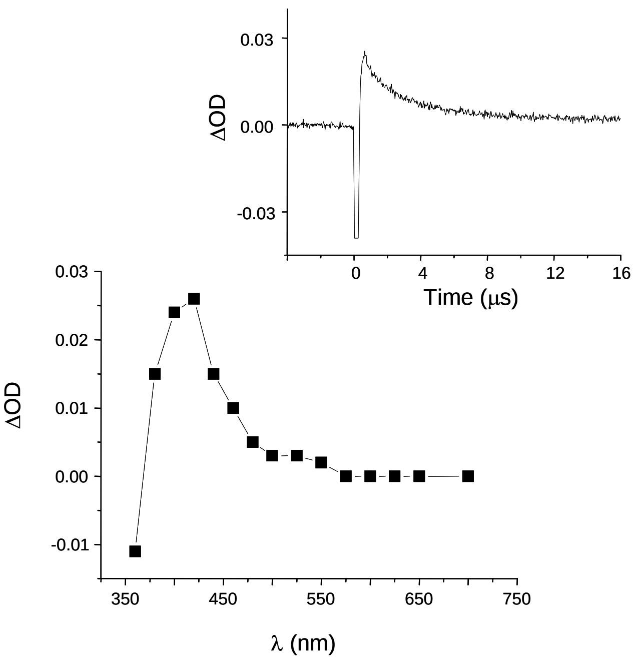


**Figure S2:** Spectrum recorded for $t$ = 1μs for the laser excitation of Py_3 at 355 nm. Insert: kinetic recorded at 420 nm.

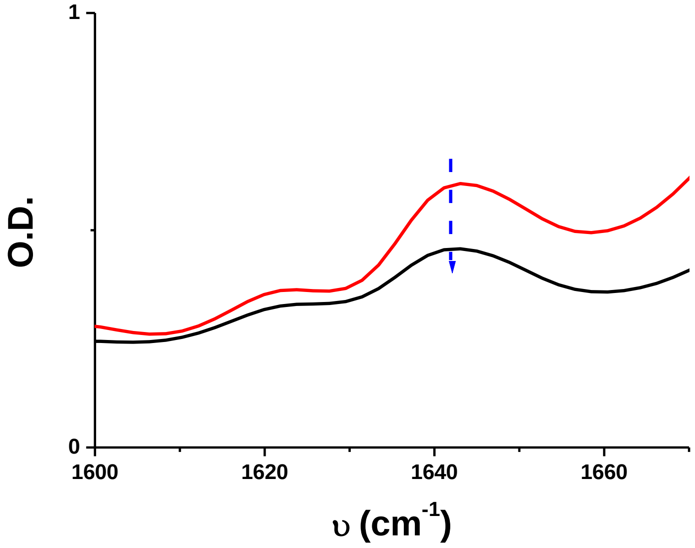


**Figure S3:** Evolution of the IR band of NVK recorded during the photopolymerization of Py_11/Iod/ NVK (0.2%/2%/3% w/w) under air and upon a halogen lamp irradiation.
